# A minimal clinically important difference measured by the Cambridge Pulmonary Hypertension Outcome Review for patients with idiopathic pulmonary arterial hypertension

**DOI:** 10.1177/2045894021995055

**Published:** 2021-05-21

**Authors:** Katherine Bunclark, Natalie Doughty, Alice Michael, Nisha Abraham, Samantha Ali, John E Cannon, Karen Sheares, Nicola Speed, Dolores Taboada, Mark Toshner, Joanna Pepke-Zaba

**Affiliations:** 1 Pulmonary Vascular Diseases Unit, Royal Papworth Hospital NHS Foundation Trust, Cambridge, UK; 2 Department of Medicine, University of Cambridge, Cambridge, UK

**Keywords:** pulmonary arterial hypertension, quality of life, health outcomes

## Abstract

Several patient-reported outcome measures have been developed to assess health status in pulmonary arterial hypertension. The required change in instrument scores needed, to be seen as meaningful to the individual, however remain unknown. We sought to identify minimal clinically important differences in the Cambridge Pulmonary Hypertension Outcome Review (CAMPHOR) and to validate these against objective markers of functional capacity. Minimal clinically important differences were established from a discovery cohort (*n* = 129) of consecutive incident cases of idiopathic pulmonary arterial hypertension with CAMPHOR scores recorded at treatment-naïve baseline and 4–12 months following pulmonary arterial hypertension therapy. An independent validation cohort (*n* = 87) was used to verify minimal clinically important differences. Concurrent measures of functional capacity relative to CAMPHOR scores were collected. Minimal clinically important differences were derived using anchor- and distributional-based approaches. In the discovery cohort, mean (SD) was 54.4 (16.4) years and 64% were female. Most patients (63%) were treated with sequential pulmonary arterial hypertension therapy. Baseline CAMPHOR scores were: Symptoms, 12 (7); Activity, 12 (7) and quality of life, 10 (7). Pulmonary arterial hypertension treatment resulted in significant improvements in CAMPHOR scores (*p* < 0.05). CAMPHOR minimal clinically important differences averaged across methods for health status improvement were: Symptoms, –4 points; Activity, –4 points and quality of life –3 points. CAMPHOR Activity score change ≥minimal clinically important difference was associated with significantly greater improvement in six-minute walk distance, in both discovery and validation populations. In conclusion, CAMPHOR scores are responsive to pulmonary arterial hypertension treatment. Minimal clinically important differences in pulmonary hypertension-specific scales may provide useful insights into treatment response in future clinical trials.

## Introduction

Pulmonary arterial hypertension (PAH) is a rare disorder characterised by a progressive rise in mean pulmonary artery pressure and pulmonary vascular resistance, ultimately resulting in right heart failure and death.^1^ PAH may present with a range of non-specific, yet debilitating symptoms which can affect health-related quality of life (HRQoL).^1–3^ As there is presently no cure for PAH, pharmacotherapy remains the mainstay of treatment with the aim of slowing disease progression and alleviating symptoms.^1^ Despite recent treatment advancements improving PAH survival, symptomatic burden remains high.^4–8^

Conventionally, establishing drug efficacy in PAH clinical trials has relied upon observed changes in functional status and capacity.^4^ It has not until relatively recently that composite morbidity and mortality end-points have been employed in event-driven trials, as highlighted by Sitbon et al.^9^ Whether selected trial end-points are relevant to the individual, however, remains unknown. As a result, there is an increasing awareness of the need for patient-reported outcomes (PROs) to be incorporated as secondary end-points in PAH clinical trials.^10^

Despite this, PROs continue to remain under-utilised. This, in part, is because changes in HRQoL measures have been more modest than objective end-points such as six-minute walk distance^11,12^ or pulmonary haemodynamics.^4^ Generic measures of HRQoL (e.g. SF-36) conventionally used in such trials, however, may lack sensitivity to detect change in specific disease processes such as PAH.^13^ To address this, a number of pulmonary hypertension-specific HRQoL instruments have been developed and validated: Cambridge Pulmonary Hypertension Outcome Review (CAMPHOR),^14^ EmPHasis-10,^2^ Living with Pulmonary Hypertension^15^ and Pulmonary Arterial Hypertension-Symptoms and Impact (PAH-SYMPACT).^16^ Such disease-specific PROs have been shown to track functional status, clinical deterioration and prognosis in PAH.^12,17^ The magnitude of improvement in these measures needed to be seen as meaningful by the individual, or the Minimal Clinically Important Difference (MCID), however, are unknown. This is of importance as even in the absence of statistically significant changes in PRO end-points, interventions may still be of relevance to the patient. Furthermore, knowledge of a measure’s MCID provides useful information regarding longitudinal changes in PROs and the monitoring of individual patient’s clinical progression.

Although the MCID has become a standard approach in the interpretation of the clinical relevance of changes in PROs, there remains no ‘gold-standard’ for MCID estimation and methodological approaches remain much debated.^18^ Broadly, MCIDs may be estimated using distributional- or anchor-based approaches.^19^ Distributional methods rely on the statistical characteristics of scores around the mean (e.g. standard deviation (SD)) whilst anchor-based methods link changes in PRO scores to a second external measure of change, or the anchor, and are therefore presumed to be sample independent. Global assessments of health change are most frequently employed as anchors in MCID estimations and enable the direct association of PRO score change to a patient’s preferences and values.^20^ They are, however, subject to recall bias.^21^ Given limitations in both distributional and anchor-based MCID methods, conventionally both methods are employed with MCIDs typically reported as the mean of combined estimates.^22^

We provide the first estimation of a MCID in a pulmonary hypertension-specific PRO measure (CAMPHOR) using both distributional- and anchor-based methods. Furthermore, we demonstrate validation of these estimates using objective markers of PAH severity.

## Methods

All incident and prevalent cases of idiopathic pulmonary arterial hypertension (IPAH) between January 2006 and June 2018 were eligible for inclusion. Follow-up was included until 1 June 2019. All patients were age >18 years at the time of diagnostic right heart catheterisation. IPAH diagnosis and treatment was as per international guideline recommendations at the time of diagnosis.^1,23,24^ Clinical data were collected prospectively into a dedicated pulmonary vascular diseases database.

The discovery cohort was comprised of all incident cases of IPAH with CAMPHOR scores available at treatment-naïve baseline and within 4–12 months following the initiation of PAH-specific therapy. A minimum follow-up of four months was chosen as this reflects the upper limit of the 12–16 week end-point historically employed for outcome assessment in PAH clinical trials.^9^ A maximum follow-up interval of 12 months was chosen as beyond this, it is increasingly difficult to attribute changes directly related to the initiation of drug therapy.

The validation dataset was formed of incident and prevalent cases of IPAH not included in the discovery cohort with at least two serial CAMPHOR scores recorded at any time point until end of follow-up. In the main, individuals in the validation cohort were prevalent cases diagnosed before the routine clinical use of CAMPHOR in 2006. Baseline pre-treatment PRO scores were therefore unavailable for this group. A minimum time period of six weeks between CAMPHOR scores was set to limit the testing effect of repeated measures completed within a short time-frame. No maximum time limit between CAMPHOR completion dates was set for the validation cohort. Prevalent cases underwent clinical review every six months as standard with CAMPHOR questionnaires completed at each visit. New York Heart Association (NYHA) functional class, six-minute walk distance (6MWD) and N-Terminal pro-Brain Natriuretic Peptide (NT-proBNP) levels concurrent to CAMPHOR completion were recorded where available.

### CAMPHOR questionnaire

The CAMPHOR questionnaire contains 65 items measuring; Symptoms (25 questions), Activity (15 questions) and quality of life (25 questions). Symptoms and quality of life are both scored out of 25, and activity out of 30. Scores are negatively weighted so that a higher score reflects worse quality of life and greater functional limitation.^14^

At the beginning of the CAMPHOR questionnaire, patients are also prompted to provide responses to two global ratings of health status. One question assesses current health status with available responses of poor/fair/good/excellent, and the other, change in health status relative to last clinical review with available responses ranging from ‘significantly worse’ to ‘significantly better’ on a seven-point scale.

### Statistical analysis

Statistical analysis was performed using R version 3.6.1.^25^ Data averages for continuous variables were reported as mean (SD) and categorical variables as n (percentage of total). As CAMPHOR scores are ordinal, values were rounded to the nearest whole number. Paired t-tests were used to compare CAMPHOR scores at baseline and post-treatment. Reported *p*-values were adjusted for multiple comparisons by false discovery rate at 5%, where necessary. Survival was calculated using a censoring date of last clinic visit or 1 June 2019, whichever was later. The National Health Service summary care record tracking system was used to ascertain survival status (searched 1 June 2019). Cox Proportional Hazards models were used to assess associations between baseline characteristics and five-year survival.

### MCID estimation

In the absence of gold-standard methodology, MCID estimates were based upon prevailing methodological approaches reported in systematic review^22^ and expert opinion.^18–21^

#### Distributional-based MCID estimation

MCIDs were estimated using three distributional-based approaches:

*SD*: the SD represents the variation among a group of scores. As 0.5-SD is widely accepted as corresponding to the MCID,^26^ this statistic was adopted for the purposes of MCID estimation in this study. The SD of all scores for each of the three CAMPHOR subdomains was divided by 2 to derive 0.5 SD.

*Effect size (ES):* ES is a standardised measure of change which can be expressed mathematically as^27,28^

$$ES=\frac{m_{2}-m_{1}}{\delta_{1}}$$

where *m*_2_ = group mean at follow-up

*m*_1_ = group mean at baseline

δ_1_ = group standard deviation at baseline 

MCIDs expressed as ES reduce bias which mainly result from dependency on baseline scores.^22^ As the MCID of a scale is generally considered to correspond to a small ES (0.2), the above formula was re-arranged so that the ES MCID was attained by multiplying the SD of baseline scores by 0.2.^29,30^

*Standard error of measurement (SEM):* the SEM for each of the three CAMPHOR subscales was derived using the calculation^28^

$$\text{SEM}=\sigma_{x}\surd 1-r_{xx}$$

where σ_x_ = the standard deviation at baseline

r_xx_ = the reliability of the HRQoL measure

Test–retest reliability coefficients for each of the three CAMPHOR subscales have been established and validated: symptoms, 0.92; activities, 0.86 and quality of life (QoL), 0.92.^14^ A number of thresholds have been suggested (1-, 1.96- and 2-SEM) when employing the SEM in MCID estimation.^31–33^ As the most widely validated is 1-SEM, this was used for the purposes of this study.^31^

#### Anchor-based MCID estimation

Anchor-based MCID estimations were attained using within-person and sensitivity-based approaches using methods similar to Van Der Roer et al.:^34^

*Within-person global ratings change*: this is the first and most widely used of the anchor-based MICID approaches.^20,22^ It defines the MCID as ‘the change in PRO scores of a group of patients selected according to their answers to a global assessment scale’ which serves as the anchor.^20^ A seven-point global rating of health status change was utilised as an anchor for this study with available ratings of ‘very much worse’ (–3) to ‘very much better’ (+3). The MCID was calculated as the mean score change from baseline to post-treatment of those who reported they were ‘moderately better’ (+2) compared to initial baseline review.

*Sensitivity and specificity analysis:* Receiver Operating Curves (ROC) were used to determine the score change from treatment-naïve baseline to post-treatment with equal sensitivity and specificity to discriminate between ‘improved’ and ‘unchanged’ patients. Improved patients were those reporting a health status change of ‘moderately better’ (+2) or ‘very much better’ (+3) compared to treatment-naïve baseline. Unchanged patients were those who reported a health status change of ‘a little worse/better’ (±1) or ‘no change’ (0) from baseline.

## Results

There were 184 consecutive incident cases of IPAH during the study period, of whom 129 patients had available pre- and post-PAH treatment CAMPHOR scores and formed the discovery cohort. The characteristics of incident IPAH patients included and excluded from the discovery cohort did not differ (online Appendix Table 1).

**Table 1.** Patient demographics and characteristics at treatment-naïve baseline and following PAH treatment (*n* = 129).

	*n*	Treatment-naïve baseline	*n*	Post-PAH treatment	Adjusted *p*
Age, years	129	54.4 (16.4)			
Sex, female %	129	63.9			
Transfer factor, % pred	83	66 (30)			
Vasoresponder^a^, *n* (%)	129	3 (2)			
PAH therapy, *n* (%)	109				
Monotherapy		69 (63)			
Dual therapy		13 (12)			
IV therapy		24 (22)			
RAP, mmHg	109	11 (13)			
Mean PAP, mmHg	109	52 (12)			
PVR, dynes/s/cm-5	109	1304 (1030)			
CO, l/min	109	3.6 (1.1)			
NYHA class, *n* (%)	122		102		
1		1 (1)		6 (6)	
2		14 (11)		29 (29)	
3		92 (75)		64 (63)	
4		15 (12)		3 (3)	0.001
CAMPHOR score					
Symptoms	129	12 (7)	129	10 (7)	0.001
Activity	129	12 (7)	129	11 (8)	0.041
Quality of life	129	10 (7)	129	9 (7)	0.009
6MWD, m	110	291 (123)	95	344 (145)	0.007
NTproBNP, pg/ml^b^	118	1154 (2286)	114	429 (1447)	0.001

Notes: Values are expressed as mean (standard deviation) unless otherwise specified. *p*-values were adjusted for multiple comparisons. Data were recorded closest to the time of IPAH diagnostic right heart catheter and before PAH treatment with pulmonary vasodilators (treatment-naïve baseline) and at 4–12 month clinical review following the initiation of PAH therapy (post PAH treatment).

PAH: pulmonary arterial hypertension; RAP: right atrial pressure; PAP: pulmonary arterial pressure; PVR: pulmonary vascular resistance; CO: cardiac output; NYHA: New York Heart Association functional class; CAMPHOR: Cambridge Pulmonary Hypertension Outcome Review questionnaire; 6MWD: six-minute walk distance; NT-proBNP: N-Terminal pro-Brain Natriuretic Peptide.

^a^Positive response to vasoreactivity challenge with nitric oxide as defined by current international guideline.^1^

^b^Values expressed as median (interquartile range).

Discovery cohort patient demographics and characteristics at treatment-naive baseline and post PAH treatment are outlined in Table 1. Mean (SD) age was 54.4 (16.4) years and 64% were female. Three individuals were vasoresponders to nitric oxide. The majority of patients (63%) were treated following PAH diagnosis with oral monotherapy (sequential therapy). Twenty-four patients (22%) were treated with upfront parenteral prostanoid therapy. In the five years following IPAH diagnosis, 32 patients in the discovery cohort died and one underwent lung transplantation.

**Fig. 1.**
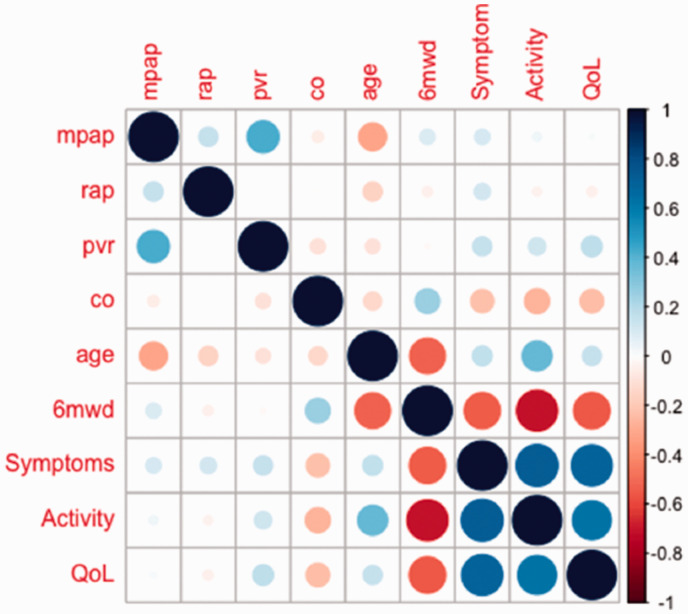
Correlation matrix for baseline CAMPHOR scores against haemodynamic, age and functional status. Positive correlations are displayed in blue and negative correlations in red. Colour intensity and the size of the circle are proportional to the correlation coefficients. mpap: mean pulmonary artery pressure; rap: right atrial pressure (mean); pvr: pulmonary vascular resistance; co: cardiac output; 6MWD: six-minute walk distance; QoL: quality of life.

CAMPHOR scores at treatment-naïve baseline were mean (SD): Symptoms, 12 (7); Activity, 12 (7) and QoL, 10 (7). Baseline CAMPHOR scores were poorly correlated with pre-PAH treatment haemodynamics (Symptoms, *r* = 0.10–0.33; Activity, *r* = 0.04–0.30; QoL, *r* = 0.02–0.23) but were moderately correlated with 6MWD (Symptoms, *r* = –0.54; Activity, *r* = –0.70; QoL, *r* = –0.55, *p* < 0.001; all, Fig. 1). Discovery cohort 1-, 3- and 5-year survival was 98.4%, 83.2% and 70.1%. Mortality was associated with a higher activity score, older age and lower six-minute walk distance at PAH diagnosis as well as female sex (Table 2).

**Table 2.** Cox proportional hazard model of survival from date of IPAH diagnosis.

	Units	HR (95% CI)	*p* Values
CAMPHOR			
Symptoms	1	1.06 (1.00–1.12)	NS
Activity	1	1.06 (1.01–1.11)	0.031
QoL	1	1.03 (0.98–1.09)	NS
Age	10	1.32 (1.05–1.66)	0.019
Sex	Female	2.26 (1.13–4.54)	0.021
6MWD	10	0.96 (0.93–0.99)	0.015
Haemodynamics			
mPAP	1	0.97 (0.94–1.01)	NS
PVR	1	1.00 (1.00–1.01)	NS
Cardiac index	1	0.57 (0.31–1.05)	NS

Notes: The association between baseline variables (treatment-naïve) and long-term survival was assessed. IPAH diagnosis date taken from time of diagnostic right heart catheterisation.

CAMPHOR: Cambridge Pulmonary Hypertension Outcome Review; QoL: quality of life; 6MWD: six-minute walk distance; mPAP: mean pulmonary artery pressure; PVR: pulmonary vascular resistance; CI: confidence interval; HR: hazard ratio.

PAH therapy was associated with a significant improvement in CAMPHOR subdomain scores: Symptoms: *p* = 0.001; Activity, *p* = 0.041; QoL, *p* = 0.009 (Table 1). Significant gains were also seen in NYHA functional class, 6MWD and plasma NT-proBNP level with treatment (*p* < 0.01; all, Table 1). Change in CAMPHOR score was weakly correlated with baseline score (i.e. higher baseline score associated with greatest score reduction): Symptoms, (Pearson) *r* = –0.34; Activities, *r* = –0.31 and QoL *r* = – 0.43 (*p* < 0.001; all).

### Global ratings of health

Patient-reported global health status at baseline (*n* = 108) was: Poor (*n* (%), 26 (24%); Fair, 44 (41%); Good, 34 (31%) and Very Good, 4 (4%). The proportion of patients reporting a Good or Very Good health status increased post-PAH treatment (*n* = 114): Poor (*n* (%)), 13 (11%), Fair, 37 (32%), Good, 47 (41%) and Very Good, 17 (15%; *p* = 0.002). Global health ratings were appropriately discriminated by CAMPHOR subscale scores: Symptoms, *p* < 0.001; Activity, *p* < 0.001; QoL, *p* = 0.012 (online Appendix Table 2).

A total of 117 individuals provided global ratings of health change following PAH treatment. The seven potential responses of ‘very much worse’ to ‘very much better’ were discriminated by CAMPHOR subscale scores (*p* < 0.001, all; Table 3). Frequencies of reported change are displayed in online Appendix Figure 1. Thirty-five individuals (30%) reported a change in health status of at least ‘moderately better’ with PAH treatment and were considered to have ‘improved’.

**Table 3.** Change in CAMPHOR score for global ratings of health status change with PAH treatment (*n* = 117).

	*n*	Symptoms	Activity	Quality of life
Very much worse	4	7 (5)	8 (5)	9 (7)
Moderately worse	8	3 (6)	3 (6)	4 (6)
A little worse	16	0 (4)	–1 (4)	0 (4)
Not changed	31	–2 (4)	–2 (4)	–1 (5)
A little better	23	–1 (7)	0 (4)	–1 (5)
Moderately better	18	–5 (5)	–4 (5)	–4 (5)
Very much better	17	–9 (6)	–4 (6)	–8 (6)

Notes: Values expressed as mean (standard deviation). Global health and CAMPHOR score change at 4–12 month post PAH treatment review relative to treatment-naive baseline.

### Anchor and distributional MCID estimates

Distributional MCIDs were calculated using 0.5SD, the ES and SEM as described above. For the Symptoms domain, distributional MCIDs were 4-points, 1-point and 2-points, respectively; for the Activity domain: 4-points, 1-point and 3-points, respectively and for QoL: 4-points, 2-points and 2-points (Table 4).

**Table 4.** CAMPHOR subscale MCID estimates for clinical improvement by distributional and anchor-based methods.

	Symptoms	Activity	QoL
Distributional			
0.5 SD	4	4	4
Effect size	1	1	2
SEM	2	3	2
Anchor-based			
Mean (SD) change	–5 (6)	–4 (5)	–4 (6)
Sensitivity analysis	– 6	–6	–1
(AUROC, 95% CI)	(0.79; 0.70–0.88)	(0.73; 0.63–0.84)	(0.80; 0.72–0.88)

SD: standard deviation; SEM: standard error of measurement; AUROC: Area under the Receiver Operating Curve; CI: confidence interval; QoL: quality of life.

Anchor-based MCIDs generated from the mean change in CAMPHOR score for those who reported feeling ‘moderately better’ post PAH treatment were: Symptoms, –5 points; Activity, –4 points and QoL, –4 points and from ROC thresholds: Symptoms, –6 points (Area Under Receiver Operating Cure (AUROC) 0.79; 95% Confidence Interval (CI): 0.70–0.88), Activity, –6 points (AUROC 0.73; 95% CI: 0.63–0.84) and QoL, –1 points (AUROC 0.80; 95% CI: 0.72–0.88).

Final MCID estimates derived by taking the mean of distributional- and anchor-based results were: Symptoms, 4 points; Activity, 4 points and QoL, 3 points. MCIDs in CAMPHOR subscale scores between treatment-naïve baseline and post-PAH treatment were achieved by 41 patients (32%) for Symptoms, 39 patients (30%) for Activity and 47 patients (36%). Seventeen patients (13%) achieved the MCID across all three scales.

### MCID validation

MCIDs were first compared against objective markers of PAH severity in the discovery cohort. The CAMPHOR Activity domain had the strongest correlation with 6MWD (Pearson r = 0.70, *p* < 0.001). Those attaining the Activity MCID had a greater change in 6MWD (82.3 (80.8) m versus 38.8 (75.3) m; *p* = 0.030) from a lower baseline 6MWD (250 (108) versus 311(125) m; *p* = 0.015) than those who did not. Activity MCID achievement was also associated with a greater fall in NTproBNP level (–1094 (1948) versus –448 (1736) pg/ml) and an increased frequency of NYHA functional class improvement (42% vs. 33%), although these did not reach statistical significance (*p* = 0.106 and *p* = 0.522, respectively).

MCID estimates were further verified in a validation dataset comprised of 1008 CAMPHOR observations with contemporaneous 6MWD measurements across 87 incident and prevalent cases of IPAH. Mean interval between observations was 188 days. The Activity scale again had the highest correlation with 6MWD: *r* = –0.58 (*p* < 0.001) of the three CAMPHOR subdomains. There were 94 instances of Activity MCID (–4 points) attainment between serial CAMPHOR observations during the study period. Change in 6MWD associated with Activity MCID attainment was 31.4 (64) m compared to –4.6 (35) m for episodes of Activity MCID non-attainment (*p* < 0.001).

## Discussion

The symptomatic burden of PAH and its effects on HRQoL are widely known.^4–8,35^ Changes in HRQoL in response to PAH treatment in the ‘real world’ setting, however, remains poorly understood. This is the first systematic study to directly assess the impact of PAH therapy on HRQoL outside of the clinical trial setting using a PH-specific PRO measure; CAMPHOR. We demonstrate significant improvements in each of the three CAMPHOR subdomains with PAH therapy, alongside improvements in objective measures of PAH treatment response: functional class, exercise capacity and NTproBNP level. Using distributional and anchor-based methods, we propose minimum thresholds of CAMPHOR score change deemed clinically relevant to individuals with PAH, or the MCID. Attainment of the Activity scale MCID (four-point change) was associated with a significantly higher increase in exercise capacity in both incident and prevalent population compared to those who failed to achieve the required change.

As with previous studies utilising CAMPHOR, we demonstrate moderate correlations between CAMPHOR subdomains and exercise capacity;^14^ the Activity subscale having the strongest relationship with six-minute walk distance (*r* = 0.70). Correlations compare favourably with those derived using generic (SF-36, *r* = 0.40–0.60) and PH-specific (PAH-SYMPACT, *r* = 0.14–0.57) PRO measures and reinforce the CAMPHOR as an excellent surrogate of functional limitation.^36–38^ Treatment-naive CAMPHOR scores were weakly correlated with diagnostic haemodynamics (*r* = 0.04–0.33) suggesting that factors beyond PAH haemodynamic severity measured at rest influence symptomatic burden. To our knowledge, this is the first study to relate haemodynamics to a pulmonary hypertension-specific PRO and reinforces similar findings from the use of generic-HRQoL measures.^37^

Whilst anecdotally, there is the perception that PAH therapies improve patient’s HRQoL, there is limited ‘real word’ data to support subjective physician experience. Although PROs have been incorporated as secondary end-points in PAH clinical trials, these have relied upon generic HRQoL measures which may be less sensitive to change in specific disease process such as PAH. This may at least partly explain why only modest changes in PRO end-points have been observed to-date.^13^ In this study, we demonstrate the significant improvement in CAMPHOR scores following initiation of PAH therapy (Symptoms: *p* = 0.001; Activity, *p* = 0.041; QoL, *p* = 0.009). Furthermore, improvements in CAMPHOR score tracked changes in objective measures of treatment response including: six-minute walk distance, NYHA functional class and plasma NTproBNP level, reflecting the ability of CAMPHOR scores to detect change and be responsive over time.

Survival from PAH diagnosis was associated with lower baseline CAMPHOR Activity score, younger age at diagnosis, female sex and greater exercise capacity. Whilst our limited sample size precluded a robust evaluation of the additional contribution of PROs to prognostication using proposed risk stratification tools, the significance of baseline CAMPHOR scores in predicting long-term survival highlights the insights that can be gained simply from patient perceptions alone.

In chronic diseases such as PAH where there is no ‘cure’, understanding clinically important change to patients becomes more relevant. To our knowledge, this is the first study to quantify a MCID in IPAH for a disease-specific PRO measure. Standardised methodology for MCID estimation has yet to be determined. Both distributional and anchor-based approaches have their limitations which have been extensively discussed elsewhere.^18,21^ Methodology in this study was based on prevailing consensus opinion. As global ratings of health change are the most commonly used measure when attempting to identify within patient change, this approach was adopted for anchor-based estimations.^18,21,22^

Across five methods (three distributional and two anchor-based), MCIDs for improvement in CAMPHOR were; Symptoms: –4 points, Activity: –4 points and QoL, –3 points. A third of incident patients achieved an MCID in at least one of the three CAMPHOR subdomains with PAH therapy. Change in CAMPHOR Activity scale score that was equivalent, or greater than, the MCID was associated with a significantly greater increase in six-minute walk distance from diagnostic baseline compared to those who did not attain the MCID threshold (82.3 m vs. 38.8 m; *p* = 0.03). Greater improvements in NTproBNP levels and NYHA functional class were also seen in those who attained the MCID although these did not reach statistical significance. As change in CAMPHOR score was only weakly associated with baseline scores, MCID thresholds should be relevant irrespective of the initial degree of HRQoL impairment.

The longitudinal relevance of CAMPHOR MCID estimates was demonstrated in an extended validation cohort comprised of 1008 instances of CAMPHOR score completion in 87 incident and prevalent cases of IPAH. Once again, a change in CAMPHOR Activity score at least equivalent to the MCID of –4 points between serial CAMPHOR measures was associated with greater gains in six-minute walk distance compared to individuals who did not report a MCID threshold change (31.4 (64) m versus –4.6 (35) m). This distance of 31 m associated with CAMPHOR Activity MCID attainment compares favourably to direct estimates of the MCID in 6MWD for PAH of 33 m, and reinforces the utility of these values in determining relevant change at the level of the individual.^37^ The validation of MCID estimates in both incident and prevalent populations enables not only a better understanding of the effects of intervention at the cohort level (e.g. when submitted to clinical trials) but provides useful insights when monitoring individual patient’s progress.

Whilst this study has a number of strengths, we acknowledge that although patient characteristics were similar to those of well-published PAH cohorts, data from this study originate from a single pulmonary hypertension centre and may therefore be subject to bias. CAMPHOR has also received criticism for being more time intensive than other available pulmonary hypertension QOL measures, however remains the most validated with adaption for use in, but not limited to, the: United States,^39^ Australia/New Zealand,^40^ Portugal,^41^ Germany,^42^ Netherlands,^43^ Poland,^44^ Brazil,^45^ Croatia,^46^ French/English Canada^47^ and Columbia.^48^ This provides plentiful opportunity for the external validation of MCID estimates to assess their robustness which is unafforded by other measures at present. Moreover, CAMPHOR is the only clinically utilised measure inclusive of a global assessment of health. As global assessments of health change enable the direct association of PRO score change to a patient’s preferences and values, the inclusion of anchor-based methodology in any MCID estimation is generally considered mandatory.^22^

One further limitation to our study is the absence of a gold standard methodology for MCID estimation. We have however aligned our methodological approach to prevailing consensus opinion and indeed have derived estimates using approaches in excess of those seen in the majority of other published MCID works, refining the accuracy of our estimates to the best available.^22^ Further work is required to determine universally accepted methods for MCID estimation which may be of relevance to our work in the future.

In conclusion, we have established MCIDs for patient-relevant clinical improvement in CAMPHOR subscale scores and demonstrate the correlation of the CAMPHOR Activity subscale to functional capacity. MCIDs in a pulmonary hypertension-specific PRO measure provides useful insights when monitoring individual patient’s progress and allows for a better understanding of the effects of intervention at the cohort level (e.g. when submitted to clinical trials).

## Supplemental Material

sj-pdf-1-pul-10.1177_2045894021995055 - Supplemental material for A minimal clinically important difference measured by the Cambridge Pulmonary Hypertension Outcome Review for patients with idiopathic pulmonary arterial hypertension Click here for additional data file. Supplemental material, sj-pdf-1-pul-10.1177_2045894021995055 for A minimal clinically important difference measured by the Cambridge Pulmonary Hypertension Outcome Review for patients with idiopathic pulmonary arterial hypertension by Katherine Bunclark, Natalie Doughty, Alice Michael, Nisha Abraham, Samantha Ali, John E Cannon, Karen Sheares, Nicola Speed, Dolores Taboada, Mark Toshner and Joanna Pepke-Zaba in Pulmonary Circulation

sj-pdf-2-pul-10.1177_2045894021995055 - Supplemental material for A minimal clinically important difference measured by the Cambridge Pulmonary Hypertension Outcome Review for patients with idiopathic pulmonary arterial hypertension Click here for additional data file. Supplemental material, sj-pdf-2-pul-10.1177_2045894021995055 for A minimal clinically important difference measured by the Cambridge Pulmonary Hypertension Outcome Review for patients with idiopathic pulmonary arterial hypertension by Katherine Bunclark, Natalie Doughty, Alice Michael, Nisha Abraham, Samantha Ali, John E Cannon, Karen Sheares, Nicola Speed, Dolores Taboada, Mark Toshner and Joanna Pepke-Zaba in Pulmonary Circulation

## References

[1] Galiè N Humbert M Vachiery J-L, et al. 2015 ESC/ERS guidelines for the diagnosis and treatment of pulmonary hypertension. Eur Heart J 2016; 37:67–119. 26320113 10.1093/eurheartj/ehv317

[2] Yorke J Corris P Gaine S, et al. EmPHasis-10: development of a health-related quality of life measure in pulmonary hypertension. Eur Respir J 2014; 43: 1106–1113. 24232702 10.1183/09031936.00127113 PMC3971119

[3] Delcroix M Howard L. Pulmonary arterial hypertension: the burden of disease and impact on quality of life. Eur Respir Rev 2015; 24:621–629. 26621976 10.1183/16000617.0063-2015 PMC9487616

[4] Ghofrani HA Galiè N Grimminger F, et al. Riociguat for the treatment of pulmonary arterial hypertension. N Engl J Med 2013; 369: 330–340. 23883378 10.1056/NEJMoa1209655

[5] Galie N Barbera JA Frost AE, et al. Initial use of ambrisentan plus tadalafil in pulmonary arterial hypertension. N Engl J Med 2015; 373: 834–844. 26308684 10.1056/NEJMoa1413687

[6] Pulido T Adzerikho I Channick RN, et al. Macitentan and morbidity and mortality in pulmonary arterial hypertension. N Engl J Med 2013; 369: 809–818. 23984728 10.1056/NEJMoa1213917

[7] Sitbon O Channick R Chin KM, et al. Selexipag for the treatment of pulmonary arterial hypertension. N Engl J Med 2015; 373: 2522–2533. 26699168 10.1056/NEJMoa1503184

[8] Yorke J Deaton C Campbell M, et al. Symptom severity and its effect on health-related quality of life over time in patients with pulmonary hypertension: a multisite longitudinal cohort study. BMJ Open Respir Res 2018; 5: e000263. 10.1136/bmjresp-2017-000263 PMC5844371 29531745

[9] Sitbon O Gomberg-Maitland M Granton J, et al. Clinical trial design and new therapies for pulmonary arterial hypertension. Eur Respir J 2019; 53: 1801908. 30545975 10.1183/13993003.01908-2018 PMC6351342

[10] McGoon MD Ferrari P Armstrong I, et al. The importance of patient perspectives in pulmonary hypertension. Eur Respir J 2019; 53:1801919. 30545977 10.1183/13993003.01919-2018 PMC6351339

[11] Favoccia C Kempny A Yorke J, et al . EmPHasis-10 score for the assessment of quality of life in various types of pulmonary hypertension and its relation to outcome. Eur J Prev Cardiol 2019; 26: 1338–1340. 30567456 10.1177/2047487318819161

[12] McCabe C Bennett M Doughty N, et al Patient-reported outcomes assessed by the CAMPHOR questionnaire predict clinical deterioration in idiopathic pulmonary arterial hypertension and chronic thromboembolic pulmonary hypertension. Chest 2013; 144: 522–530. 23430021 10.1378/chest.12-2443 PMC4694098

[13] Twiss J McKenna S Ganderton L, et al. Psychometric performance of the CAMPHOR and SF-36 in pulmonary hypertension. BMC Pulm Med 2013; 13: 45. 23844640 10.1186/1471-2466-13-45 PMC3751055

[14] McKenna SP Doughty N Meads DM, et al. The Cambridge Pulmonary Hypertension Outcome Review (CAMPHOR): a measure of health-related quality of life and quality of life for patients with pulmonary hypertension. Qual Life Res 2006; 15: 103–115. 16411035 10.1007/s11136-005-3513-4

[15] Bonner N Abetz L Meunier J, et al. Development and validation of the living with pulmonary hypertension questionnaire in pulmonary arterial hypertension patients. Health Qual Life Outcomes 2013; 11: 161. 24088389 10.1186/1477-7525-11-161 PMC3852970

[16] McCollister D Shaffer S Badesch DB, et al. Development of the Pulmonary Arterial Hypertension-Symptoms and Impact (PAH-SYMPACT®) questionnaire: a new patient-reported outcome instrument for PAH. Respir Res 2016; 17: 72. 27301413 10.1186/s12931-016-0388-6 PMC4908719

[17] Wort SJ Favoccia C Kempny A, et al emPHasis-10 score predicts mortality in patients with pulmonary hypertension. Eur Respir J 2018; 52: OA273.

[18] Copay AG Subach BR Glassman SD, et al. Understanding the minimum clinically important difference: a review of concepts and methods. Spine J 2007; 7: 541–546. 17448732 10.1016/j.spinee.2007.01.008

[19] Guyatt GH Osoba D Wu AW, et al. Methods to explain the clinical significance of health status measures. Mayo Clin Proc 2002; 77: 371–383. 11936935 10.4065/77.4.371

[20] Jaeschke R Singer J Guyatt GH. Measurement of health status. Ascertaining the minimal clinically important difference. Control Clin Trials 1989; 10: 407–415. 2691207 10.1016/0197-2456(89)90005-6

[21] Crosby RD Kolotkin RL Williams RG. Defining clinically meaningful change in health-related quality of life. J Clin Epidemiol 2003; 56: 395–407. 12812812 10.1016/s0895-4356(03)00044-1

[22] Jayadevappa R Cook R Chhatre S. Minimal important difference to infer changes in health-related quality of life – a systematic review. J Clin Epidemiol 2017; 89: 188–198. 28676426 10.1016/j.jclinepi.2017.06.009

[23] Galiè N, Torbicki A, Barst R, et al. Guidelines on diagnosis and treatment of pulmonary arterial hypertension: the Task Force on Diagnosis and Treatment of Pulmonary Arterial Hypertension of the European Society of Cardiology. Eur Heart J 2004; 25: 2243–2278. 15589643 10.1016/j.ehj.2004.09.014

[24] Galie N Hoeper MM Humbert M, et al. Guidelines for the diagnosis and treatment of pulmonary hypertension: The Task Force for the Diagnosis and Treatment of Pulmonary Hypertension of the European Society of Cardiology (ESC) and the European Respiratory Society (ERS), endorsed by the International Society of Heart and Lung Transplantation (ISHLT). Eur Heart J 2009; 30: 2493–2537. 10.1093/eurheartj/ehp297 19713419

[25] R Core Team *R: a language and environment for statistical computing*. Vienna, Austria: R Foundation for Statistical Computing, 2019.

[26] Norman GR Sloan JA Wyrwich KW. The truly remarkable universality of half a standard deviation: confirmation through another look. *Expert Rev* *Pharmacoecon Outcomes Res* 2004; 4: 581–585. 10.1586/14737167.4.5.581 19807551

[27] Kazis LE Anderson JJ Meenan RF. Effect sizes for interpreting changes in health status. Med Care 1989; 27: S178–S189. 2646488 10.1097/00005650-198903001-00015

[28] Wyrwich KW Wolinsky FD. Identifying meaningful intra-individual change standards for health-related quality of life measures. J Eval Clin Pract 2000; 6: 39–49. 10807023 10.1046/j.1365-2753.2000.00238.x

[29] Cohen J. Statistical power analysis for the behavioral sciences (revised ed.) New York, NY: Academic Press, 1988.

[30] Samsa G Edelman D Rothman ML, et al. Determining clinically important differences in health status measures: a general approach with illustration to the Health Utilities Index Mark II. Pharmacoeconomics 1999; 15: 141–155. 10351188 10.2165/00019053-199915020-00003

[31] Wyrwich KW Nienaber NA Tierney WM, et al. Linking clinical relevance and statistical significance in evaluating intra-individual changes in health-related quality of life. Med Care 1999; 37: 469–478. 10335749 10.1097/00005650-199905000-00006

[32] Ware J Kosinski M Keller S. *SF-36 physical and mental health summary scales: a users manual*. Boston, MA: Health Assessment Lab, New England Medical Center, 1994.

[33] McHorney CA Tarlov AR. Individual-patient monitoring in clinical practice: are available health status surveys adequate? Qual Life Res 1995; 4: 293–307. 7550178 10.1007/BF01593882

[34] Van Der Roer N Ostelo RWJG Bekkering GE, et al. Minimal clinically important change for pain intensity, functional status, and general health status in patients with nonspecific low back pain. Spine (Phila Pa 1976) 2006; 31: 578–582. 16508555 10.1097/01.brs.0000201293.57439.47

[35] Swetz KM Shanafelt TD Drozdowicz LB, et al. Symptom burden, quality of life, and attitudes toward palliative care in patients with pulmonary arterial hypertension: results from a cross-sectional patient survey. J Heart Lung Transpl 2012; 31: 1102–1108. 10.1016/j.healun.2012.08.010 22975100

[36] Mathai SC Suber T Khair RM, et al. Health-related quality of life and survival in pulmonary arterial hypertension. Ann Am Thorac Soc 2016; 13: 31–39. 26492065 10.1513/AnnalsATS.201412-572OC PMC4722843

[37] Mathai SC Puhan MA Lam D, et al. The minimal important difference in the 6-minute walk test for patients with pulmonary arterial hypertension. Am J Respir Crit Care Med 2012; 186: 428–433. 22723290 10.1164/rccm.201203-0480OC PMC3443803

[38] Chin KM Gomberg-Maitland M Channick RN, et al. Psychometric validation of the Pulmonary Arterial Hypertension-Symptoms and Impact (PAH-SYMPACT) questionnaire: results of the SYMPHONY trial. Chest 2018; 154: 848–861. 29705220 10.1016/j.chest.2018.04.027

[39] Gomberg-Maitland M Thenappan T Rizvi K, et al. United States validation of the Cambridge Pulmonary Hypertension Outcome Review (CAMPHOR). J Heart Lung Transpl 2008; 27: 124–130. 10.1016/j.healun.2007.10.004 18187098

[40] Ganderton L Jenkins S McKenna SP, et al. Validation of the Cambridge Pulmonary Hypertension Outcome Review (CAMPHOR) for the Australian and New Zealand populatio n. Respirology 2011; 16: 1235–1240. 21810146 10.1111/j.1440-1843.2011.02030.x

[41] Reis A Twiss J Vicente M, et al. Portuguese validation of the Cambridge pulmonary hypertension outcome review (CAMPHOR) questionnaire. Health Qual Life Outcomes 2016; 14: 110. 27460644 10.1186/s12955-016-0513-8 PMC4962538

[42] Cima K Twiss J Speich R, et al. The German adaptation of the Cambridge Pulmonary Hypertension Outcome Review (CAMPHOR). Health Qual Life Outcomes 2012; 10: 110. 22971041 10.1186/1477-7525-10-110 PMC3492159

[43] Wapenaar M Twiss J Wagenaar M, et al. Adaptation and validation of the Cambridge Pulmonary Hypertension Outcome Review (CAMPHOR) for the Netherlands. Neth Heart J 2016; 24: 417–424. Erratum in: *Neth Heart J* 2018; 26: 579. 27197970 10.1007/s12471-016-0849-z PMC4887309

[44] Małaczynska-Rajpold K Smukowska-Gorynia A Heaney A, et al. The Polish adaptation of the Cambridge Pulmonary Hypertension Outcome Review (CAMPHOR). Cardiol J 2020; 27: 608–615. 30338844 10.5603/CJ.a2018.0119 PMC8078996

[45] Correa RA, Pereira MC, Bizzi MF, et al. Adaptation and validation of the quality of life assessment of the Cambridge Pulmonary Hypertension Outcome Review (CAMPHOR) for Brazil. *J Patient Rep Outcomes* 2020; 4: 43. 10.1186/s41687-020-00209-6 PMC7275099 32504261

[46] Hećimović A Heaney A McKenna SP, et al. Adaption and validation of the Cambridge Pulmonary Hypertension Outcome Review (CAMPHOR) for Croatia. Acta Clin Croat 2019; 58: 3–12. 31363319 10.20471/acc.2019.58.01.01 PMC6629211

[47] Coffin D Duval K Martel S, et al. Adaptation of the Cambridge Pulmonary Hypertension Outcome Review (CAMPHOR) into French-Canadian and English-Canadian. Can Respir J 2008; 15: 77–83. 18354747 10.1155/2008/767126 PMC2677839

[48] Villaquirán C Moreno S Dueñas R, et al. Cross-cultural adaptation of the Cambridge Pulmonary Hypertension Outcome Review for use in patients with pulmonary hypertension in Colombia. J Bras Pneumol 2019; 45: e20180332. 31365733 10.1590/1806-3713/e20180332 PMC6715158

