# Supplementary material for: A minimal clinically important difference measured by the Cambridge Pulmonary Hypertension Outcome Review for patients with idiopathic pulmonary arterial hypertension
Source: Pulm Circ. 2021 May 21;11(2):2045894021995055. doi: 10.1177/2045894021995055 (PMC8149778; doi:10.1177/2045894021995055)
Supplement: sj-pdf-1-pul-10.1177_2045894021995055 - Supplemental material for A minimal clinically important difference measured by the Cambridge Pulmonary Hypertension Outcome Review for patients with idiopathic pulmonary arterial hypertension [file sj-pdf-1-pul-10.1177_2045894021995055.pdf]

Supplemental Material Figure 1: Frequencies of global health change following PAH treatment (n = 117)

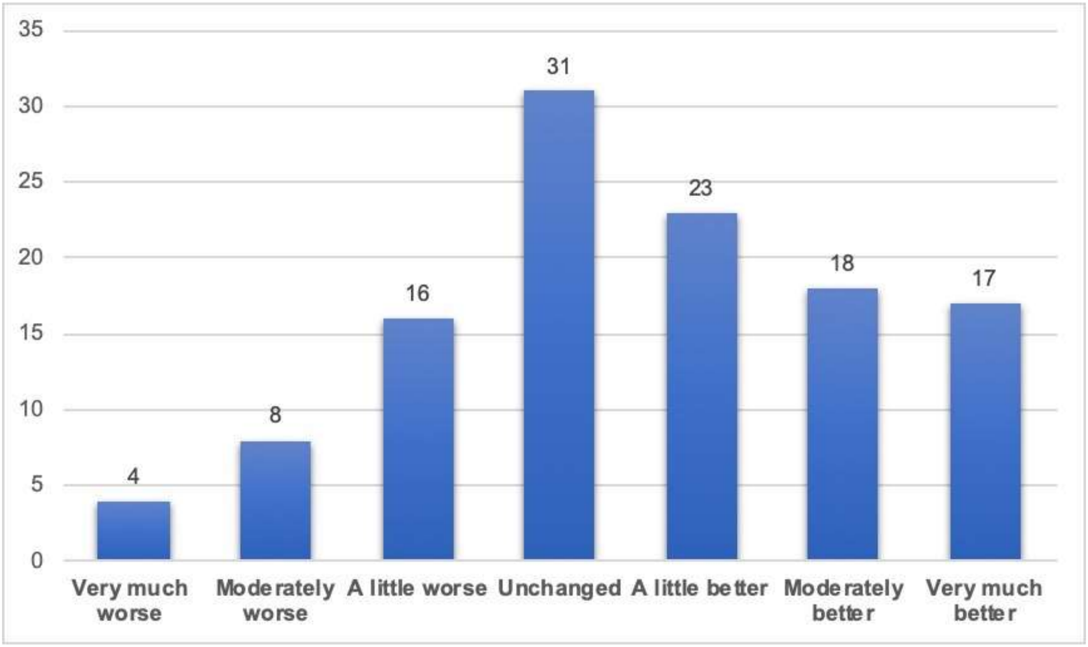

Patient self-report of change in health status from treatment-naïve baseline to 6 – 9 months post pulmonary vasodilator therapy initiation in individuals with Idiopathic PAH

PAH: Pulmonary Arterial Hypertension
