# Supplementary material for: A minimal clinically important difference measured by the Cambridge Pulmonary Hypertension Outcome Review for patients with idiopathic pulmonary arterial hypertension
Source: Pulm Circ. 2021 May 21;11(2):2045894021995055. doi: 10.1177/2045894021995055 (PMC8149778; doi:10.1177/2045894021995055)
Supplement: sj-pdf-2-pul-10.1177_2045894021995055 - Supplemental material for A minimal clinically important difference measured by the Cambridge Pulmonary Hypertension Outcome Review for patients with idiopathic pulmonary arterial hypertension [file sj-pdf-2-pul-10.1177_2045894021995055.pdf]

Supplemental Material Table 1: Patient characteristics of incident cases of IPAH included (n = 129) and excluded (n = 55) from MCID estimation

	n	**Discovery cohort**	n	**Excluded patients**
Age, years	129	54.4 (16.4)	55	57.3 (27.7)
Sex, female %	129	63.9	55	70.9
RAP, mmHg	109	10.8 (13.4)	40	10.2 (5.1)
Mean PAP, mmHg	109	51.5 (11.6)	40	51.4 (11.5)
PVR, dynes	109	1304 (1030)	39	1214 (702)
CO, l/min	109	3.6 (1.1)	39	3.9 (1.9)
CAMPHOR score				
Symptoms	129	12 (7)	42	13 (7)
Activity	129	12 (7)	42	12 (8)
Quality of Life	129	10 (7)	42	12 (7)
6MWD, metres	110	291 (123)	33	274 (126)

Values expressed as mean (standard deviation). Patients excluded from MCID estimation due to the absence of CAMPHOR scale scores available for comparison at treatment-naïve baseline and post-PAH treatment.

IPAH: Idiopathic pulmonary arterial hypertension; MCID: Minimal Clinically Important Difference; CAMPHOR: Cambridge Pulmonary Hypertension Outcome Review Questionnaire.

Supplemental Material Table 2: CAMPHOR subscale scores for global rating of health status (n = 129)

	n	**Symptoms**	**Activity**	**Quality of Life**
Poor	39	17 (5)	18 (6)	16 (5)
Fair	81	14 (5)	14 (6)	12 (6)
Good	81	7 (5)	7 (5)	5 (5)
Very Good	21	2 (2)	3 (2)	2 (2)

Values expressed as mean (standard deviation). Each individual provided global health ratings at treatment-naïve baseline and post PAH treatment. Total missing observations n = 36.
CAMPHOR: Cambridge Pulmonary Hypertension Outcome Review
